# Regulation of the PI3K/AKT Pathway and Fuel Utilization During Primate Torpor in the Gray Mouse Lemur, *Microcebus murinus*

**DOI:** 10.1016/j.gpb.2015.03.006

**Published:** 2015-06-17

**Authors:** Shannon N. Tessier, Jing Zhang, Kyle K. Biggar, Cheng-Wei Wu, Fabien Pifferi, Martine Perret, Kenneth B. Storey

**Affiliations:** 1 Institute of Biochemistry & Department of Biology, Carleton University, Ottawa, ON K1S 5B6, Canada; 2 UMR 7179 Centre National de la Recherche Scientifique, Muséum National d’Histoire Naturelle, Brunoy 91800, France; 3 Department of Surgery & Center for Engineering in Medicine, Massachusetts General Hospital & Harvard Medical School, Charlestown, MA 02129, USA; 4 Chemistry and Chemical Engineering Department, Royal Military College of Canada, Kingston, ON K7K 7B4, Canada; 5 Biochemistry Department, Schulich School of Medicine and Dentistry, Western University, London, ON N6A 5C1, Canada; 6 Department of Biology, Genetics Institute, University of Florida, Gainesville, FL 32611, USA

**Keywords:** Insulin signaling pathway, PI3K/AKT, mTOR, GSK3, Pyruvate dehydrogenase, Metabolic rate depression

## Abstract

Gray mouse lemurs (*Microcebus murinus*) from Madagascar present an excellent model for studies of torpor regulation in a primate species. In the present study, we analyzed the response of the insulin signaling pathway as well as controls on carbohydrate sparing in six different tissues of torpid versus aroused gray mouse lemurs. We found that the relative level of phospho-insulin receptor substrate (IRS-1) was significantly increased in muscle, whereas the level of phospho-insulin receptor (IR) was decreased in white adipose tissue (WAT) of torpid animals, both suggesting an inhibition of insulin/insulin-like growth factor-1 (IGF-1) signaling during torpor in these tissues. By contrast, the level of phospho-IR was increased in the liver. Interestingly, muscle, WAT, and liver occupy central roles in whole body homeostasis and each displays regulatory controls operating at the plasma membrane. Changes in other tissues included an increase in phospho-glycogen synthase kinase 3α (GSK3α) and decrease in phospho-ribosomal protein S6 (RPS6) in the heart, and a decrease in phospho-mammalian target of rapamycin (mTOR) in the kidney. Pyruvate dehydrogenase (PDH) that gates carbohydrate entry into mitochondria is inhibited via phosphorylation by pyruvate dehydrogenase kinase (*e.g.*, PDK4). In the skeletal muscle, the protein expression of PDK4 and phosphorylated PDH at Ser 300 was increased, suggesting inhibition during torpor. In contrast, there were no changes in levels of PDH expression and phosphorylation in other tissues comparing torpid and aroused animals. Information gained from these studies highlight the molecular controls that help to regulate metabolic rate depression and balance energetics during primate torpor.

## Introduction

Seasonal hibernation and daily torpor are fascinating phenomena, whereby animals enter a state of hypometabolism during which most body functions are strongly suppressed [1]. Daily torpor occurs in many species of mammals and seasonal hibernation is found in mammalian groups including monotremes, marsupials, rodents, bats, shrews, insectivores, and bears [1]. Among them, rodents, such as ground squirrels and hamsters, have been the main model species for most lab-based studies [2]. Recent studies of the small lemur species of Madagascar have discovered that these lemurs also use torpor and can even enter multi-day hibernation in response to chronic food shortages during the dry season [3]. These small cheirogaleids, which include dwarf lemurs (*Cheirogaleus*) and mouse lemurs (*Microcebus*), represent the only family among the primates with members capable of entering torpor/hibernation bouts [4]. The lemur model is extremely attractive for biomedical studies of hypometabolism, since these primates are the most closely-related species to man that exhibit natural hypometabolism and they often do so at relatively high body temperatures, indicating that primate torpor does not necessarily require prolonged cold body temperatures. Indeed, data collected from free-ranging gray mouse lemurs have shown that daily torpor bouts are several hours in duration with minimum *T*_b_ of ∼27 °C, whereas hibernation bouts can last up to 4 weeks with minimum *T*_b_ of 11.5 °C [4]. Similar to other forms of hibernation, prolonged hibernation bouts in gray mouse lemurs are characterized by intermittent arousals back to normothermic *T*_b_ [4]. The molecular mechanisms displayed by lemurs for torpor entry/arousal could reveal important information about how to induce and regulate a torpor state in humans, a goal of interest for a variety of purposes, such as organ preservation and long-term space flight.

**Figure 1:**
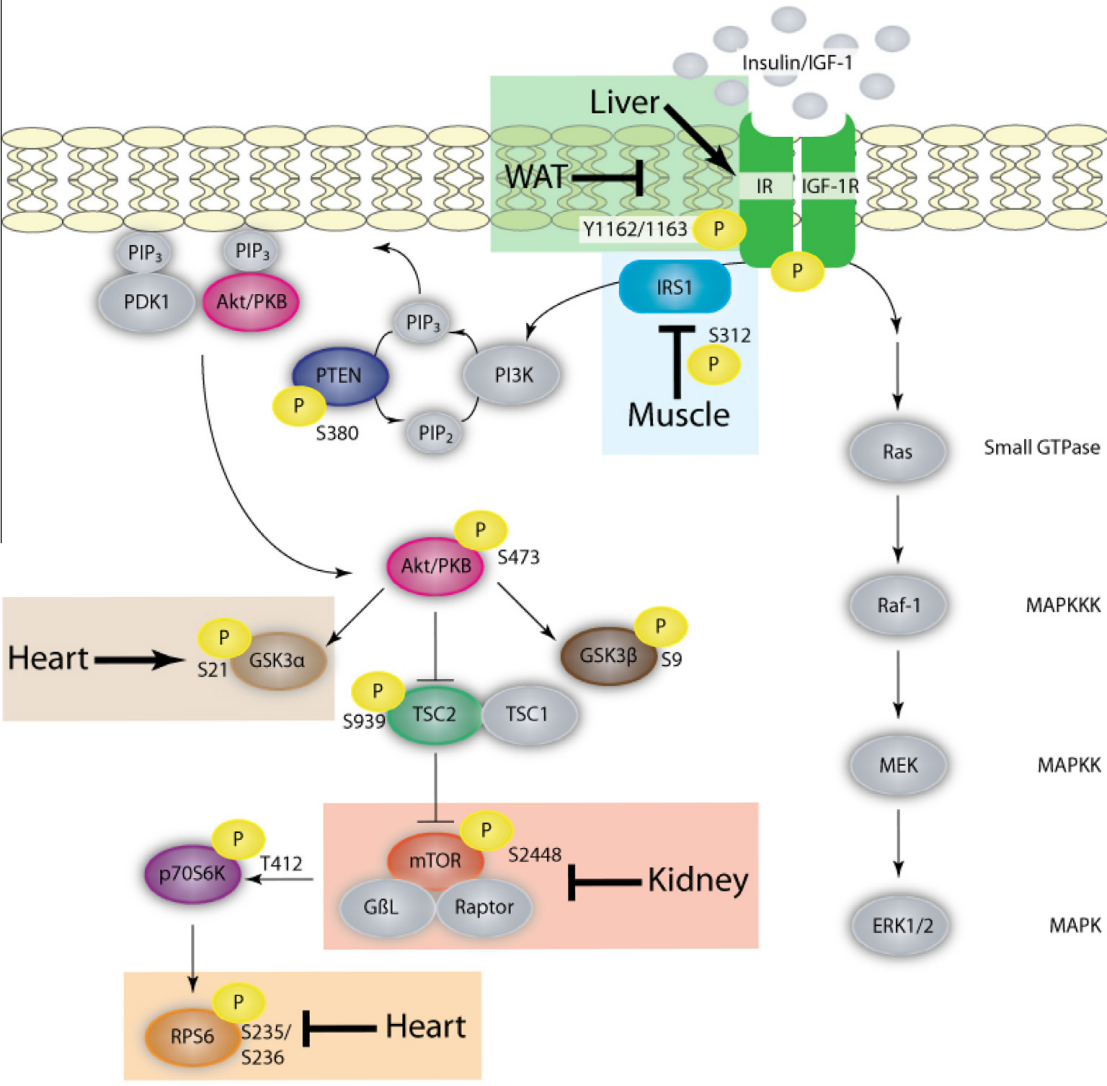
**Schematic representation of insulin and IGF-1 signaling in gray mouse lemurs** IR and IGF-IR share a similar signaling pathway which invokes downstream changes in two main branches – the mitogenic pathway (Ras/Raf/MEK/ERK) and the metabolic pathway (PI3K/Akt). These act in a coordinated manner to regulate glucose, lipid, and protein metabolism. The targets that exhibited relative changes between control and torpid conditions in each tissue are boxed and the targets examined are highlighted in associated colors (targets depicted in gray were not examined in the present study). Arrowheads denote positive regulatory effects while blunt-ended lines denote negative regulatory effects. Shaded boxes designate the tissue-specific response observed in gray mouse lemur, *Microcebus murinus*, during torpor. Akt is also known as Protein Kinase B (PKB). IR, insulin receptor; IGF-1R, insulin-like growth factor-1; IRS1, insulin receptor substrate 1; PI3K, phosphoinositide 3-kinase; PIP3, phosphatidylinositol triphosphate; PTEN, phosphatase and tensin homolog; PDK1, phosphoinositide-dependent protein kinase-1; GSK3, glycogen synthase kinase 3; TSC, tuberous sclerosis protein; mTOR, mammalian target of rapamycin; GβL, G-protein β-subunit-like protein; p70S6K, p70S6 kinase; RPS6, ribosomal protein S6; MEK, mitogen-activated protein kinase kinase, ERK1/2, extracellular-signal-regulated kinases 1/2; WAT, white adipose tissue.

One common theme in the field of hibernation is the involvement of signal transduction pathways, which restructure cellular metabolism to promote energy conservation and survival. The Akt pathway regulates events including apoptosis, protein synthesis, cell proliferation, and energy metabolism, and thereby lies at a junction between metabolism and cellular survival [5–7]. Akt (also known as Protein Kinase B) is the core kinase in the insulin/Akt signaling network and plays a noted role in aerobic dormancy [7]. The activation of Akt is dependent on multi-site phosphorylation, which is driven by a series of kinase-based signal transduction events. Binding of insulin/insulin-like growth factor-1 (IGF-1) to receptor tyrosine kinases, insulin receptor (IR) or IGF-1 receptor (IGF-1R), gates the signal to insulin receptor substrate 1 (IRS1), phosphatidylinositol (3,4,5)-trisphosphate kinase (PI3K), and eventually Akt (Figure 1). The direct upstream kinase responsible for stimulating Akt kinase activity is phosphoinositide-dependent kinase-1 (PDK1). To promote the phosphorylation of Akt by PDK1, both proteins are anchored to the plasma membrane by phosphatidylinositol trisphosphate (PIP3) [8,9]. PIP3 is generated by PI3K-dependent phosphorylation of PIP2 and the reverse reaction is regulated by the phosphatase PTEN (phosphatase and tensin homolog) [10,11]. The activated form of Akt influences protein synthesis via mammalian target of rapamycin (mTOR) as well as glucose metabolism via glycogen synthase kinase 3 (GSK3). Akt signaling activates the mTOR pathway via phosphorylation of tuberous sclerosis protein 2 (TSC2), thereby repressing its inhibitory activity on mTOR complex 1 (mTORC1) assembly [12]. Active mTORC1 in turn phosphorylates p70S6K, which then phosphorylates eukaryotic initiation factor 4B (eIF4B) and ribosomal protein S6 (RPS6), which are directly involved in the formation of the mRNA translation pre-initiation complex (Figure 1) [13,14].

In anticipation of the winter season, seasonal mammalian hibernators enter a phase of hyperphagia resulting in weight gains of up to 40% as they fatten [2,15]. This is matched with a sharp shift in the hibernator’s metabolic profile, which moves from oxidation of carbohydrates toward a dependence on the combustion of stored fatty acids [16]. While lipids are the primary fuel source during hibernation, rates of mitochondrial substrate oxidation and oxidative phosphorylation are nonetheless strongly reduced as part of overall metabolic rate suppression [17]. Key factors in achieving the suppression of mitochondrial activity are mechanisms which gate carbohydrate entry into the tricarboxylic acid cycle and impose an overall suppression of oxidative phosphorylation [17]. Pyruvate dehydrogenase (PDH), which converts pyruvate to acetyl-CoA, is strongly inhibited via phosphorylation at multiple sites by pyruvate dehydrogenase kinase isoforms, including PDK4 [18]. Key regulatory phosphorylation sites on PDH include Ser 232, 293, and 300. In the meadow jumping mice (*Zapus hudosnius*), the percentage of PDH in the active form dropped from 15% in the heart and 29% in the kidney of euthermic animals to just 1% in animals that had been hibernating for 5–8 days [17]. In thirteen-lined ground squirrels, compared to the summer active state, gene expression of *PDK4* increased in skeletal muscle and white adipose tissue (WAT), together with increased amounts of PDK4 protein in the heart, skeletal muscle, and WAT of hibernating animals [19]. In line with the increased PDK expression, other studies have also shown that the amount of activated, dephosphorylated PDH in the heart and kidney of torpid animals fell to just 3%−4% of euthermic squirrels [20]. These data suggest that both PDH and PDK4 play important roles in fuel utilization and metabolic depression during torpor.

Although the behavioral and physiological adaptations supporting torpor in gray mouse lemurs have been well studied, the molecular mechanisms supporting daily torpor have yet to be fully elucidated. Since the insulin/IGF-1 pathway and PDH/PDK4 are central regulators of metabolism and survival, we investigated the phosphorylation status of key factors involved in these processes. Our results demonstrate that insulin/Akt signaling networks and PDH control are integral components of the hypometabolic state in lemur tissues.

## Results

### Response of Akt/mTOR signaling during daily torpor

The insulin/Akt signaling pathway is regulated by posttranslational modifications such as protein phosphorylation at multiple distinct sites, which are indicative of the activity state of the target protein. As a result, antibodies that recognize these phosphorylation sites were used to monitor changes in the activity state of components of the insulin/Akt signaling pathway comparing control (aroused) and torpid conditions in gray mouse lemurs. To evaluate the response of Akt/mTOR pathway to daily torpor, the relative changes in levels of phosphorylated proteins were assessed in different tissues, including the skeletal muscle, heart, liver, kidney, brown adipose tissue (BAT), and white adipose tissue (WAT), using commercially-available multiplex panels. The phosphoproteins examined included IGF-1R (Tyr1135/Tyr1136), IR (Tyr1162/Tyr1163), IRS1 (Ser312), PTEN (Ser380), Akt (Ser473), GSK3α (Ser21), GSK3β (Ser9), TSC2 (Ser939), mTOR (Ser2448), p70S6K (Thr412), and RPS6 (Ser235/Ser236).

**Figure 2:**
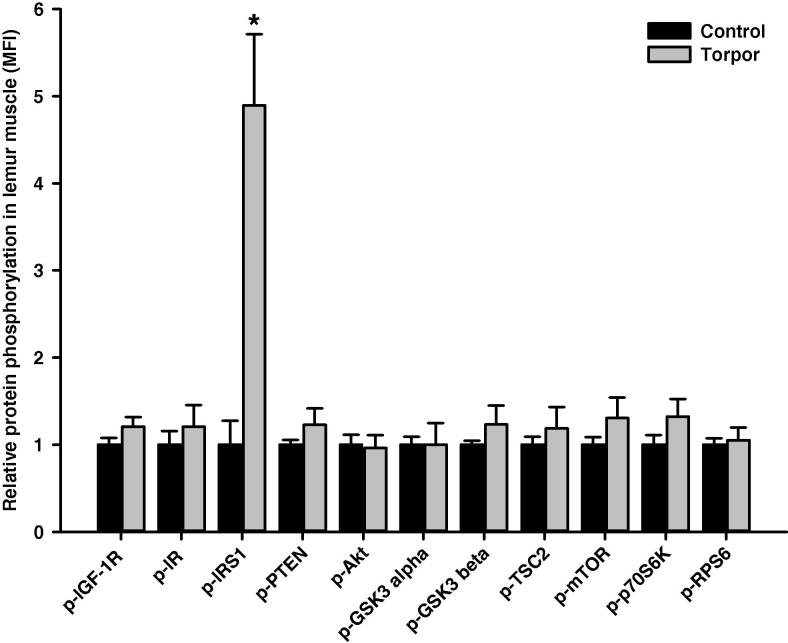
**Responses of Akt/mTOR signaling to daily torpor in skeletal muscle of gray mouse lemurs** The relative phosphorylation of multiple target proteins in the skeletal muscles was assessed for IGF-1R (Tyr1135/Tyr1136), IR (Tyr1162/Tyr1163), IRS1 (Ser312), PTEN (Ser380), Akt (Ser473), GSK3α (Ser21), GSK3β (Ser9), TSC2 (Ser939), mTOR (Ser2448), p70S6K (Thr412), and RPS6 (Ser235/Ser236) comparing control (aroused) and torpor states. The relative MFI for a given protein in a sample was calculated by normalizing all samples to controls. Data are presented as mean ± SEM (*n* = 4 independent protein isolations from different animals). * Denotes significant difference from the corresponding control according to the two-tailed Student’s *t*-test (*P *< 0.01). MFI, median fluorescent intensity.

**Figure 3:**
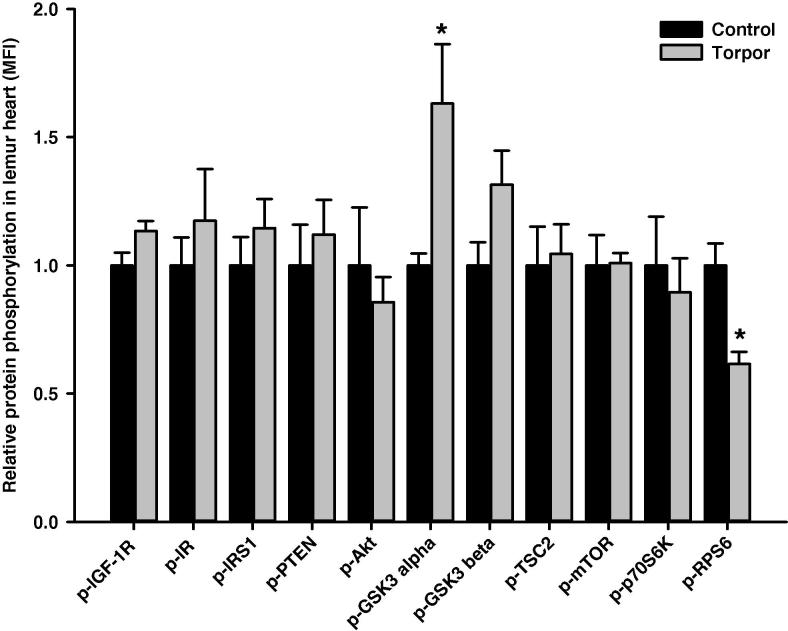
**Responses of Akt/mTOR signaling to daily torpor in heart of gray mouse lemurs** The relative phosphorylation of multiple target proteins in heart was assessed for IGF-1R (Tyr1135/Tyr1136); IR (Tyr1162/Tyr1163), IRS1 (Ser312), PTEN (Ser380), Akt (Ser473), GSK3α (Ser21), GSK3β (Ser9), TSC2 (Ser939), mTOR (Ser2448), p70S6K (Thr412), and RPS6 (Ser235/Ser236) comparing control (aroused) and torpor states. Data were obtained and analyzed similarly as indicated in Figure 2. * Denotes significant difference from the corresponding control according to the two-tailed Student’s *t*-test (*P *< 0.05).

**Figure 4:**
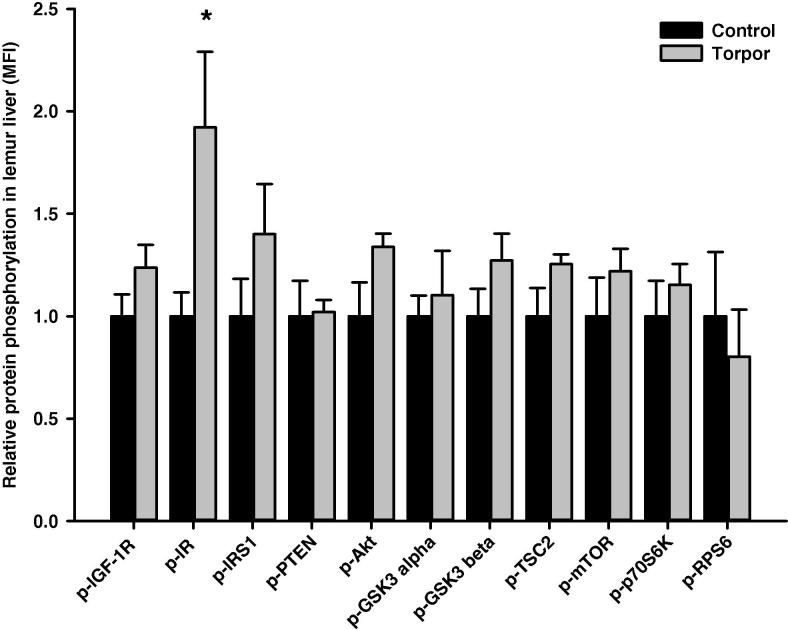
**Responses of Akt/mTOR signaling to daily torpor in liver of gray mouse lemurs** The relative phosphorylation of multiple proteins in liver was assessed for IGF-1R (Tyr1135/Tyr1136); IR (Tyr1162/Tyr1163), IRS1 (Ser312), PTEN (Ser380), Akt (Ser473), GSK3α (Ser21), GSK3β (Ser9), TSC2 (Ser939), mTOR (Ser2448), p70S6K (Thr412), and RPS6 (Ser235/Ser236) comparing control (aroused) and torpor states. Data were obtained and analyzed similarly as indicated in Figure 2. * Denotes significant difference from the corresponding control according to the two-tailed Student’s *t*-test (*P *< 0.05).

**Figure 5:**
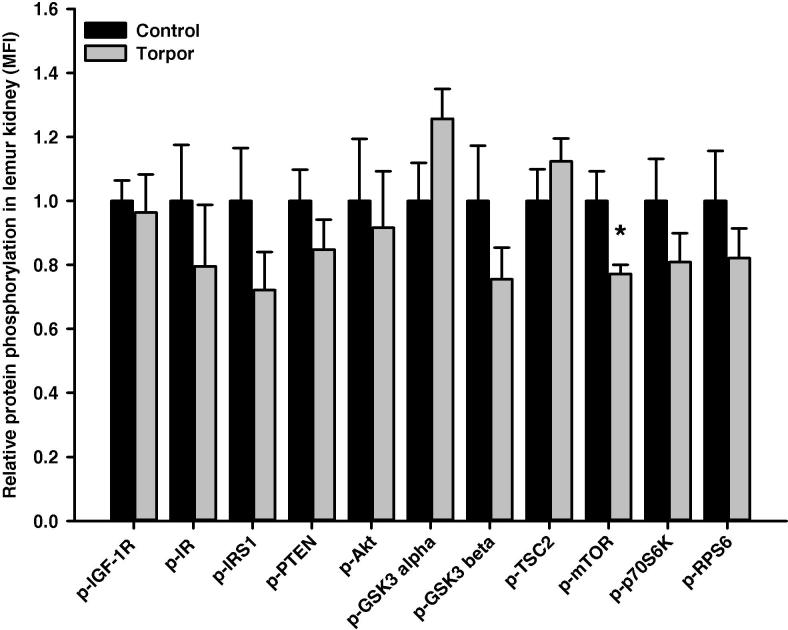
**Responses of Akt/mTOR signaling to daily torpor in kidney of gray mouse lemurs** The relative phosphorylation of multiple proteins in kidney was assessed for IGF-1R (Tyr1135/Tyr1136); IR (Tyr1162/Tyr1163), IRS1 (Ser312), PTEN (Ser380), Akt (Ser473), GSK3α (Ser21), GSK3β (Ser9), TSC2 (Ser939), mTOR (Ser2448), p70S6K (Thr412), and RPS6 (Ser235/Ser236) comparing control (aroused) and torpor states. Data were obtained and analyzed similarly as indicated in Figure 2. * Denotes significant difference from the corresponding control according to the two-tailed Student’s *t*-test (*P *< 0.05).

In the skeletal muscle, relative protein level of IRS1 (Ser312) was significantly higher during torpor, which is 4.89 ± 0.82-fold higher than in control aroused animals (*P *< 0.01), whereas phosphorylation levels of all the other targets remained unchanged (Figure 2). Among the 11 proteins examined, we noticed significant alterations in two of them in the heart. Levels of GSK3α (Ser21) in torpid animals was 1.63 ± 0.23-fold of that in controls, whereas levels of RPS6 (Ser235/236) in the torpid lemurs was reduced, which was only 61 ± 5% of that in aroused animals (*P *< 0.05) (Figure 3). In the liver, the relative phosphorylation level of IR (Tyr1162/1163) during torpor was 1.92 ± 0.37-fold of that in control animals (*P *< 0.05), whereas relative phosphorylation levels were comparable for the other proteins (Figure 4). In the kidney, the relative phosphorylation of most proteins except GSK3 alpha appeared to be lower during torpor. However, significant alteration during torpor was only detected in the level of mTOR (Ser2448), which was reduced to 77 ± 3% of controls (*P < *0.05) (Figure 5).

**Figure 6:**
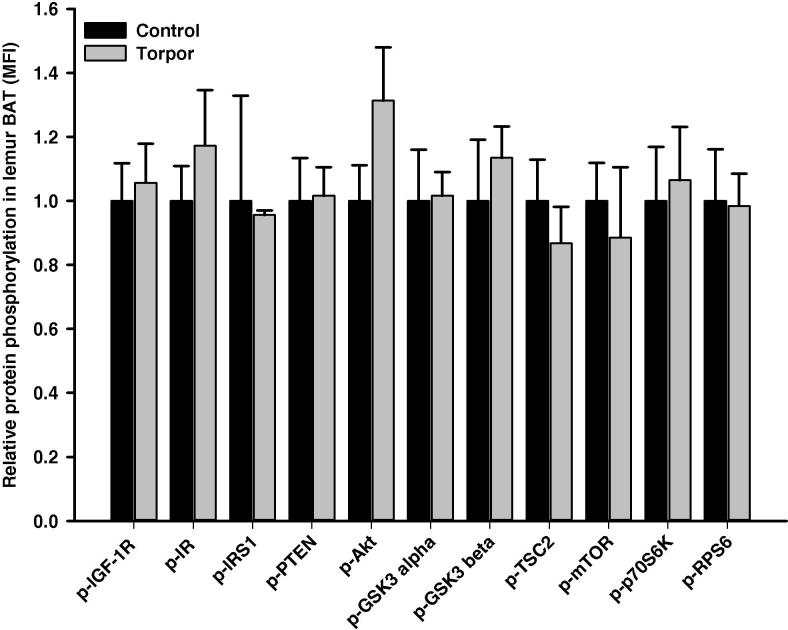
**Responses of Akt/mTOR signaling to daily torpor in BAT of gray mouse lemurs** The relative phosphorylation of multiple proteins in BAT was assessed for IGF-1R (Tyr1135/Tyr1136); IR (Tyr1162/Tyr1163), IRS1 (Ser312), PTEN (Ser380), Akt (Ser473), GSK3α (Ser21), GSK3β (Ser9), TSC2 (Ser939), mTOR (Ser2448), p70S6K (Thr412), and RPS6 (Ser235/Ser236) comparing control (aroused) and torpor states. Data were obtained and analyzed similarly as indicated in Figure 2. BAT, brown adipose tissue.

**Figure 7:**
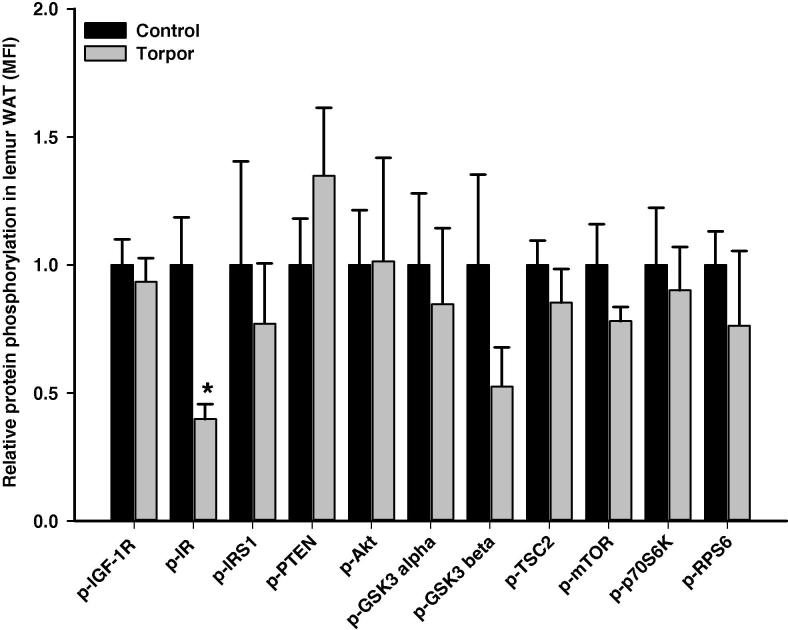
**Responses of Akt/mTOR signaling to daily torpor in WAT of gray mouse lemurs** The relative phosphorylation of multiple proteins in WAT was assessed for IGF-1R (Tyr1135/Tyr1136); IR (Tyr1162/Tyr1163), IRS1 (Ser312), PTEN (Ser380), Akt (Ser473), GSK3α (Ser21), GSK3β (Ser9), TSC2 (Ser939), mTOR (Ser2448), p70S6K (Thr412), and RPS6 (Ser235/Ser236) comparing control (aroused) and torpor states. Data were obtained and analyzed similarly as indicated in Figure 2. * Denotes significant difference from the corresponding control according to the two-tailed Student’s *t*-test (*P *< 0.05). WAT, white adipose tissue.

Interestingly, torpor seems to have no significant effect on any of the targets in BAT (Figure 6). However, the relative phosphorylation of IR (Tyr1162/1163) in WAT was significantly reduced during torpor (40 ± 6% of controls; *P < *0.05) (Figure 7), which is opposite to the response seen in the liver. No significant changes in the relative levels of phosphorylation were detected for other proteins examined in WAT during torpor.

### Response of PDK4 and PDH during daily torpor

**Figure 8:**
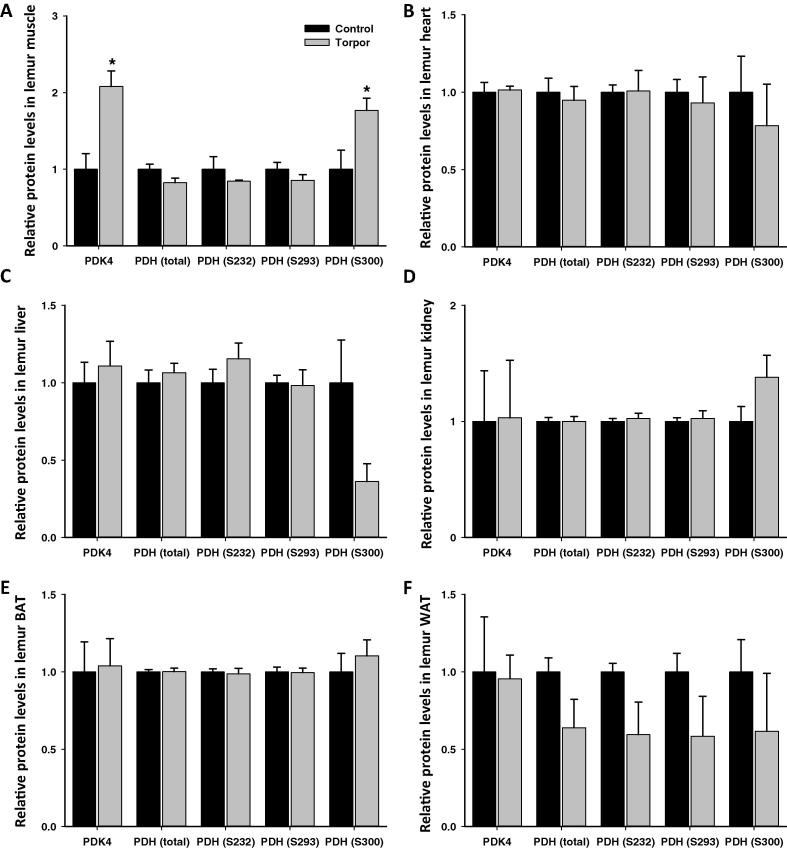
**Response of PDK4 and PDH to daily torpor in different tissues of gray mouse lemurs** The relative protein expression of PDH and phosphorylation of PDH at Ser232, Ser293, and Ser300) were assessed using bead-based assays and represented as relative MFI by comparing control (aroused) and torpor states. In addition, the expression of PDK4 was examined with ELISA using optical density as readout. The tissues examined include skeletal muscle (**A**), heart (**B**) liver (**C**), kidney (**D**), BAT (**E**), and WAT (**F**). Data were analyzed similarly as indicated in Figure 2. * Denotes significant difference from the corresponding control according to the two-tailed Student’s *t*-test (*P *< 0.05). MFI, median fluorescent intensity; BAT, brown adipose tissue; WAT, white adipose tissue; PDH, pyruvate dehydrogenase; PDK, pyruvate dehydrogenase kinase.

Phosphorylation on PDH is indicative of its activity state, which is inversely correlated with activity. Similarly, we examined the relative changes in total PDH protein, as well as PDH phosphorylation state at Ser 232, 293, and 300, in various tissues using commercially available multiplex panels. In addition, protein levels of PDK4, one of the kinases that phosphorylate PDH, were also assessed using ELISA technology. Compared to aroused animals, the relative levels of PDK4 and phospho-PDH during torpor were significantly (*P *< 0.05) changed only in the skeletal muscle, but comparable in the other five tissues examined (Figure 8). In skeletal muscle of torpid lemurs, relative levels of PDK4 and phospho-PDH Ser 300 were 2.08 ± 0.20-fold and 1.77 ± 0.16-fold of those in controls, respectively.

## Discussion

For many small mammals, short-term daily torpor and long-term hibernation can conserve huge amounts of energy that would otherwise be needed to maintain active euthermic life under conditions where food availability is greatly reduced and/or abiotic conditions are severe [1]. Regulatory controls on metabolism and cellular survival mediated by signal transduction pathways are at the core of the hibernating phenotype. As a result, it is no surprise that insulin/Akt signaling and PDK4/PDH regulatory mechanisms play a documented role in metabolic rate depression [7,17,21]. Indeed, reduced insulin/IGF-1 signaling correlates with dauer in *Caenorhabditis elegans* [22], diapause in insects [23], and hibernation in ground squirrels [24–27] and bats [28]. Similarly, carbohydrate sparing via regulatory controls on PDH has been demonstrated in mice [17] and ground squirrels [19,20]. Ultimately, minimizing ATP expenditures is of central importance and, as a result, stress-responsive regulation is dependent on energetically-efficient mechanisms such as reversible protein phosphorylation (RPP). Indeed, RPP-dependent regulation has been reported in a variety of stress-tolerant species ranging from invertebrates to mammals [29,30]. The present study is the first to demonstrate the involvement of insulin/Akt signaling and PDK4/PDH during torpor in a nonhuman primate, the gray mouse lemur.

Akt signaling is responsive to a range of extracellular stimuli including insulin and IGF-1 through their corresponding receptors, IR and IGF-1R, respectively [31,32]. Binding of insulin/IGF-1 triggers a cascade of intracellular events beginning with receptor tyrosine phosphorylation; key residues include phosphorylation at tyrosine 1158/1162/1163 in IR or tyrosine 1131/1135/1136 in IGF-1R, which is indicative of enhanced activity [33,34]. The activated receptor phosphorylates substrates such as IRS1, which dock downstream effector molecules spanning a range of signaling pathways [35]. Through its SH2 (Src Homology 2) domain, IRS1 makes a physical interaction with phosphotyrosine-containing regions of RTK and this interaction is abolished by phosphorylation of IRS1 at Ser312 [36,37]. Our results showed strong elevated (∼5-fold) phosphorylation of IRS1 at Ser312 in skeletal muscle during torpor (Figure 2), suggesting inhibition of insulin- and IGF-1-dependent signal transduction in torpid animals, as compared to aroused lemurs. Inhibition of insulin signaling was also observed in WAT, where the tyrosine phosphorylation level (Tyr1162/Tyr1163) of IR dropped significantly to below 50% of the control values (Figure 7).

While the inhibitory signals in muscle and WAT agree with the general metabolic pattern of reduced insulin signaling during hypometabolism, an opposing response was observed in liver. Relative level of phospho-IR (Tyr1162/Tyr1163) was almost doubled in the liver during torpor (Figure 4), suggesting activation of insulin-dependent signaling in this organ. Liver plays an essential role in providing oxidizable substrates, not only for its own needs, but also to supply those of other tissues. In hepatocytes, an increase in insulin-specific signaling is correlated with stimulation of glycogen synthesis and/or inhibition of gluconeogenesis [38]. However, gluconeogenesis is typically required for survival during prolonged fasting [39] and plays a noted role in mammalian models of hibernation [40]. Since the metabolic effects of insulin signaling are typically exerted via IRS1, further studies are needed to delineate the direct downstream effect of IR-dependent activation in the liver.

In muscle, the inactivation of IRS1 in torpid gray mouse lemurs should lead to decreased glucose uptake, glycogen synthesis, and/or rates of glycolysis. Evidence for reduced glycolytic flux and glycogen synthesis exists in mammalian hibernators [20] and this response may contribute to overall energy savings during torpor. A possible mechanism for increased phosphorylation of IRS1 may occur via c-Jun NH_2_-terminal kinase (JNK), which has been shown to be involved in phosphorylation of IRS1 on Ser312 [41]. Indeed, our studies have shown that the active form of JNK is enhanced in lemur skeletal muscle during torpor, representing a possible molecular mechanism for IRS1 inactivation [42].

Insulin signaling was also inhibited in WAT of gray mouse lemurs as indicated by a reduction in the phosphorylation state of the insulin receptor. Hibernators accumulate large reserves of triglycerides in WAT to fuel winter survival and also increase the proportion of polyunsaturated fatty acids to keep lipids fluid at low *T*_b_ [43,44]. Gray mouse lemurs also rely on fatty acid fuels for energy for daily torpor during food restriction [45]. Since inactivation of insulin signaling in WAT is correlated with decreased lipid synthesis and increased lipolysis [46,47], the data showing inhibition of insulin signaling in lemur WAT suggest that these same events occur during primate torpor. Evidence of a shift in fuel utilization was also indicated for muscle from the relative changes in PDK4 and phospho-PDH Ser 300 (Figure 8). Relative increases in these two targets during torpor, as compared to aroused animals, suggest that carbohydrate catabolism by mitochondria is suppressed. By contrast, changes in PDK4 and PDH were not observed in other lemur tissues studied. This suggests that other mechanisms which control fuel utilization or other phosphorylation sites/posttranslational modifications not presently analyzed may play a more prominent role in regulating carbohydrate versus lipid fuel use during torpor.

Interestingly, regulatory controls when present at the receptor level occur on IR rather than IGF-1R. While an array of genetic and environmental factors are thought to play roles in lifespan extension, crucial genes that are involved in modulating lifespan have been identified, including insulin receptors. Both *C. elegans* and *Drosophila melanogaster* have single IGF-1/insulin-like receptors (DAF-2 in *C. elegans* and INR in *D. melanogaster*), and several homologs (IGF-1R, IR-A, and IR-B) are present in mammals [48,49]. *daf-2* mutant showed extended lifespan, indicating that insulin/IGF-1 antagonizes longevity [50]. Gray mouse lemurs are exceptionally long-lived, whose lifespans are 2–3 times longer than other mammals of comparable body mass [51]. However, the molecular basis for this response remains largely unexplored. In the present study, inhibitory signals such as phosphorylation of insulin receptors in selected tissues of gray mouse lemurs may be a point of interest for further studies. Indeed, the use of lemur models in the field of life extension is already well documented [52].

Despite upstream changes, the relative phosphorylation levels of the downstream targets measured remained unaltered in muscle, WAT, and liver during torpor. This suggests that IR/IRS1 signaling during torpor may not be mediated by Akt-dependent signal propagation. While insulin/IR and IGF-/IGF-1R are strong activators of PI3K-mTOR, these signals also regulate Ras-ERK mitogen-activated protein kinase (MAPK) signal transduction (Figure 1), albeit to a lesser degree [53]. IR and IGF-1R are connected to Ras-ERK signaling via a direct interaction with the Shc:Grb2:SOS complex as well as with IRS1 [35]. We have demonstrated a significant decrease in the phosphorylation of ERK1/2 in muscle of torpid lemurs [42]. Therefore, it is possible that the regulation of IRS1 observed in the present study links to Ras-ERK signaling rather than to Akt-mTOR signaling. In the same study, however, the relative phosphorylation level of ERK1/2 increased in WAT and remained unaltered in the liver [42]. Since the degree of pathway activation depends on a combination of factors, further studies are required to elucidate the full downstream impact of IR/IRS1 signaling on muscle, WAT, and liver.

One well-known role of Akt is the phosphorylation-dependent regulation of GSK3 [54,55]. Our results showed that the phosphorylation level of GSK3α at Ser21 was elevated significantly in the heart of torpid animals as compared to controls (Figure 3) with probable inhibitory effects on multiple targets of GSK3 during torpor. For example, GSK3 inhibits glycogen synthesis by phosphorylation of glycogen synthase [55]. GSK3 is also involved in regulating other major biological events. It was reported that GSK3 negatively regulates cyclin E by promoting its degradation and this subsequently leads to inhibition of cell cycle progression [56]. GSK3α plays an essential role in β-adrenergic signaling as well, which is directly related to cardiac function in mammals, and participates in maintaining mitochondrial structure in the heart [57].

The mTOR pathway has a major role in regulating cap-dependent translation [58–60] and mTOR-dependent protein synthesis regulation is dependent on the assembly and function of the kinase complex mTORC1. The mTORC1-dependent regulation on translation initiation is in part controlled through p70S6K. Active mTORC1 phosphorylates p70S6K and, in turn, activated p70S6K phosphorylates RPS6 [13,14]. Torpid lemurs showed a significant reduction in the phosphorylation level of RPS6 (Ser235/Ser236) in the heart compared with aroused animals (Figure 3), suggesting inhibition of protein synthesis during torpor. Interestingly, the phosphorylation level of mTOR at Ser2448 dropped significantly in the kidney of torpid lemurs when compared to control animals (Figure 5). Hence, both the heart and the kidney showed signs of inhibition of the mTOR pathway during torpor, indicating protein synthesis repression. These results agree with the general energy conservation strategy of hypometabolic states.

In summary, while tissue-specific responses were observed, insulin/IGF1-dependent signal transduction was inhibited in muscle and WAT through regulatory events occurring on IRS1 and IR, respectively. As discussed above, inhibition of insulin signaling in muscle and WAT may act to balance the energy production-consumption equilibrium during the hypometabolic state. In contrast, activation of insulin-dependent signal transduction was indicated in the liver. However, the outcome may be Akt-independent in liver, since specific nodes of the Akt pathway failed to exhibit signs of regulation. In both the heart and the kidney, daily torpor led to inhibition of the mTOR pathway, suggesting repression of protein synthesis. In addition, elevation of GSK3α phosphorylation in the heart may contribute to the coordinated suppression of mitochondrial respiration during primate torpor. Finally, the protein expression of PDK4 increased significantly and this was matched with a significant increase in the phosphorylation of PDH at Ser300, suggesting the conversion of pyruvate to acetyl-CoA is significantly reduced in muscle during torpor. In conclusion, the present study provides insights into the regulation of PI3K/Akt signaling and fuel utilization in six tissues of the gray mouse lemur during torpor. Further studies may include carrying out deeper transcriptomic and proteomic analysis.

## Materials and methods

### Animals

Standard procedures for holding, experimentation and sampling of gray mouse lemurs were used and all animal experiments were conducted by Dr. Martine Perret and the MECADEV team (Mecanismes Adaptatifs et Evolution, Department of Ecology and Management of Biodiversity) as described by Giroud et al. [3] and presented in detail by Biggar and his colleagues [42]. Briefly, adult female lemurs were housed in individual cages in a climate chamber, where they were maintained under short-day conditions and held at a thermoneutral ambient temperature (24–25 °C). Animals in the torpor group had been exposed to a calorie-restricted diet for 5 days (60% of the control diet; 86 × 10^−3^ J/day versus 144 × 10^−3^ J/day) to enhance the depth of their torpor bouts. Control animals were capable of entering torpor and were fed *ad libitum*. *T*_b_ and locomotion were monitored and used to determine the state of torpor, which was assessed as a continuous reduction in *T*_b_ (with no evidence of animal activity). Control animals were euthanized after arousal from a daily torpor bout (after spontaneous rewarming to 35–36 °C), whereas torpid lemurs were euthanized during a torpor bout (when *T*_b_ was at its minimum, 30–33 °C). Samples of frozen tissues were packed in dry ice and air freighted to Carleton University where they were stored continuously at −80 °C until use.

### Protein lysates

Protein extracts of tissue samples were prepared as per manufacturer’s instructions (EMD Millipore, Billerica, MA; catalog No. 48-611). Briefly, ∼50 mg aliquots of frozen tissue were weighed and homogenized 1:4 (w/v) with ice-cold lysis buffer (Millipore; catalog No. 43-040) in a Dounce homogenizer with the further addition of phosphatase (1 mM Na_3_VO_4_ and 10 mM ß-glycerophosphate) and protease (BioShop; catalog No. PIC001) inhibitors. Samples were then incubated on ice for 30 min with occasional vortexing. Homogenates were centrifuged at 12,000 × *g* for 20 min at 4 °C and the supernatants were collected as total soluble protein lysates. Protein concentration of the lysates was determined using the Bradford assay (Bio-Rad; catalog No. 500-0005) and then tissue extracts were standardized to 5 μg/μl and stored at −80 °C until further use.

### Multiplex analysis

Luminex® assays were used to investigate the relative phosphorylation state of key components involved in insulin/Akt signal transduction (EMD Millipore; catalog No. 48-611) and PDH (EMD Millipore; catalog No. PDHMAG-13K) in the muscle, heart, liver, kidney, BAT, and WAT comparing control (aroused) and torpid mouse lemurs. The assay for the insulin/Akt Luminex® panel was performed following manufacturer’s instructions and all multiplex panels are subject to rigorous quality control and validation studies. Aliquots of protein homogenates (5 μg/μl) were combined with Milliplex MAP Assay Buffer 2 (catalog No. 43-041), with 17.5 μg total protein added per well. HeLa cell lysates treated with the dual specificity lambda phosphatase (catalog No.47-229) were used as a negative control, while positive controls included insulin-stimulated HepG2 cells (catalog No. 47-227) and MCF7 cells stimulated with IGF-1 (catalog No. 47-216). The same protocol was followed for the PDH panel except for the use of Assay Buffer 1 (catalog No. 43-010) with 10.5 μg protein added per well. For the PDH kit, HepG2 cell lysates catalog No. 47-234) were used as a positive control, whereas HepG2 cell lysates treated with dichloroacetate (DCA) (catalog No. 47-232), a PDK inhibitor, served as a negative control. Positive and negative controls were prepared as per manufacturer’s instructions.

For the Akt panel, premixed phosphoprotein beads (catalog No. 42-611K) for all the protein targets were provided as a 20 × stock, which were sonicated for 15 s, vortexed for 30 s, diluted to 1 ×, and vortexed once more for 15 s. After calibration with Assay Buffer, the bead mixture was gently mixed with a pipette and sonicated for 10 s and then 25 μl 1 × phosphoprotein beads was added to each well. Following the addition of the phosphoprotein beads, equal amounts of diluted cell lysate was added to each sample well, whereas HeLa, HepG2, and MCF7 cell lysates were added to control wells. The sample and control wells were incubated overnight at 4 °C on a plate shaker (600–800 rpm) protected from light. After removal of the lysate by vacuum filtration, all wells were washed twice with Assay Buffer 2 (catalog No. 43-041). Afterward, 25 μl 1 × biotin-labeled detection antibodies was added to each well and the filter plate was incubated on a plate shaker for 1 h at room temperature. Following removal of the antibody solution by vacuum filtration, the filter plate was washed twice (as above). Streptavidin–phycoerythrin (25 ×, SAPE, catalog No. 45-001D) was then diluted in Assay Buffer and aliquoted into wells (25 μl). Following incubation on a plate shaker for 15 min, 25 μl of Amplification Buffer (catalog No. 43-024A) was added to each well and incubated for 15 min. The SAPE/Amplification Buffer was then removed by vacuum filtration. The beads were resuspended in 150 μl Assay Buffer 2 and data acquisition was performed on a Luminex 100 instrument (Luminex, Austin, TX) with Milliplex Analyst software (Millipore, Billerica, MA). Equipment settings were as follows: 50 events per bead, sample size of 100 μl, and gate settings of 8000–15,000.

The PDH kit used magnetic beads (EMD Millipore; catalog No. PDHMAG-PMX4), which were provided as a 1 × stock. Beads were sonicated for 30 s and vortexed, and 25 μl was added to each well following the addition of 25 μl each of Assay Buffer 1, lysate controls, or samples. The plate was incubated on a plate shaker (600–800 rpm) protected from light for 2 h at room temperature. The well contents were removed and the plate was washed 3 times using a magnetic plate washer. Afterward, 50 μl of detection antibody was added and incubated on a plate shaker at room temperature for 1 h before the washing step. Next, 50 μl SAPE was added to each well, incubated on a plate shaker at room temperature for 30 min. Finally, the beads were resuspended in 100 μl Sheath Fluid after wash and data acquisition was performed as described above.

### Enzyme-linked immunosorbent assay

The relative protein expression of PDK4 was determined using a BlueGene Elisa kit (catalog No. E01P0080) purchased from Life Sciences Advanced Technologies (Saint Petersburg, FL) following the manufacturer’s instructions. Aliquots of 50 μl of sample were added to wells of a microtiter plate that were pre-coated with antibodies, whereas negative control wells received 50 μl of phosphate-buffered saline (pH 7.2). Next, 5 μl of the supplied balance solution was added to each sample, followed by 100 μl of supplied antibody conjugate. Samples were covered and incubated for 1 h at 37 °C before the wells were washed with the supplied 1 × wash buffer (5 times). Next, 50 μl each of substrate A and substrate B were added to each well, covered, and incubated for 15 min at room temperature in the dark. Finally, 50 μl of stop solution was added to each well and the optical density at 450 nm was determined using a Thermoscan microplate reader.

### Statistical analysis

Bead- and ELISA-based assays used the median fluorescence intensity (MFI) and mean absorbance, respectively, to determine the relative protein levels. All numerical data are expressed as mean ± SEM (*n* = 4) normalized to control (aroused) values. Statistical analysis was performed using SigmaPlot statistical package (v.12) software. The two-tailed Student’s *t*-test was employed to assess differences between samples from aroused and torpid animals and difference was considered significant with *P *< 0.05 or *P *< 0.01.

## Authors’ contributions

All authors contributed to the conception and design of the project and to the editing of the manuscript. MP and FP carried out the animal experiments; SNT, JZ, KKB, and CWW conducted biochemical assays. Data analysis and assembly of the draft manuscript were carried out by SNT, JZ, and KBS. All authors read and approved the final manuscript.

## Competing interests

The authors have declared no competing interests.
